# The effect of reduced carbohydrate diet on cardiometabolic risk factors and gut microbiota in children and adolescents with obesity: a randomized controlled trial

**DOI:** 10.1186/s12887-026-07516-9

**Published:** 2026-08-15

**Authors:** Yasaman Khorshidi, Farzad Hadaegh, Nazgoli Rafiei, Parto Hadaegh, Shadab Salehpour, Hanieh-Sadat Ejtahed, Atoosa Saidpour, Golaleh Asghari

**Affiliations:** 1 Department of Clinical Nutrition and Dietetics, Faculty of Nutrition Sciences and Food Technology, Shahid Beheshti University of Medical Sciences, P.O. Box 19395-4741, Tehran, Iran; 2 Prevention of Metabolic Disorders Research Center, Research Institute for Endocrine Sciences, Shahid Beheshti University of Medical Sciences, Tehran, Iran; 3 Department of Pediatrics, Shahid Beheshti University of Medical Sciences, Tehran, Iran; 4 Obesity and Eating Habits Research Center, Endocrinology and Metabolism Clinical Sciences Institute, Tehran University of Medical Sciences, Tehran, Iran; 5 Endocrinology and Metabolism Research Center, Endocrinology and Metabolism Clinical Sciences Institute, Tehran University of Medical Sciences, Tehran, Iran

**Keywords:** Children, Adolescents, Obesity, Reduced carbohydrate diet, Cardiometabolic, Gut microbiota

## Abstract

**Objective:**

To compare the effects of a reduced-carbohydrate diet (RCD) with a standard diet (STD) on cardiometabolic risk factors and gut microbiota composition in children and adolescents with obesity.

**Methods:**

In this randomized controlled trial, 84 children and adolescents with obesity, aged 7–16 years, were randomly assigned to either a RCD (30–40% of total energy from carbohydrates, emphasizing high‑fiber sources) or a STD for 12 weeks. A calorie deficit was applied in both groups. Dietary assessments, anthropometric measurements, body composition analysis, biochemical measurements, and gut microbiota composition were evaluated at baseline and at the end of follow-up. Change in body mass index at 12 weeks was considered the primary outcome.

**Results:**

Sixty-one participants (72.6%) completed the intervention. Both the reduced-carbohydrate diet (RCD) and standard diet (STD) groups showed significant reductions in BMI, BMI z-score, waist circumference, and percent body fat over the 12-week intervention, with no significant differences between groups (*p* > 0.05).Triglyceride levels and the triglyceride-to-HDL cholesterol ratio decreased significantly in the RCD group compared with the STD group (between-group mean difference − 40.31 mg/dL, 95% CI − 73.79 to − 6.82, *p* = 0.019 for TG; − 1.03, 95% CI − 1.85 to − 0.20, *p* = 0.016 for TG/HDL-C). The relative abundance of Verrucomicrobia increased in the RCD group, whereas it decreased in the STD group.

**Conclusions:**

The reduced carbohydrate diet did not show superiority to standard diet in reducing anthropometric indices. However, reduced carbohydrate diet showed beneficial effects on Triglyceride levels and Triglycerides to high-density lipoprotein cholesterol ratio. Further investigation of gut microbiota composition is needed.

**Trial registration:**

Iranian Registry of Clinical Trials (IRCT), IRCT20180805040703N2, retrospectively registered on 7 May 2022.

**Supplementary Information:**

The online version contains supplementary material available at https://doi.org/10.1186/s12887-026-07516-9.

## Introduction

The prevalence of obesity among children and adolescents has reached epidemic proportions over the past decade and represents a major global public health challenge [1]. Childhood obesity is associated with numerous adverse health outcomes. Excess adiposity, particularly in the abdominal region, contributes to insulin resistance (IR) and chronic low‑grade inflammation, which may lead to metabolic syndrome, non‑alcoholic fatty liver disease, type 2 diabetes, and cardiovascular disease during childhood and later in adulthood [2, 3]. Moreover, insulin resistance may further promote adiposity, creating a vicious cycle that exacerbates metabolic dysfunction [4]. Obesity is a multifactorial condition resulting from complex interactions between genetic and environmental factors, including dietary habits. A sustained positive energy balance due to sedentary behavior and consumption of energy-dense processed foods is considered a primary driver of childhood obesity [5, 6]. More recently, the insulin–carbohydrate model has been proposed as an alternative explanation, suggesting that the intake of rapidly digestible carbohydrates promotes fat deposition through insulin-mediated hormonal pathways [7]. This model has generated both support and debate. Some researchers propose that hormonal mechanisms, including increased insulin secretion and altered energy partitioning, may contribute to obesity development and metabolic dysregulation [8]. However, experimental studies have challenged several predictions of this model, indicating that it may not fully explain obesity pathogenesis [9]. In pediatric populations, evidence regarding carbohydrate modification remains limited and inconsistent. While some systematic reviews report modest improvements in insulin sensitivity, clinical guidelines emphasize the lack of conclusive evidence in children and adolescents [10, 11]. Beyond carbohydrate quantity, carbohydrate quality defined by glycemic index, glycemic load, fiber content, and simple sugar content also plays a crucial role in metabolic health [12, 13].

The gut microbiota has emerged as an important regulator of energy homeostasis and metabolic function. Significant differences in gut microbiota composition have been observed between children with obesity and those with normal weight [14–16]. Diet is one of the most important modifiable determinants of gut microbiota composition. Diets high in fat, sugar, and energy density may alter microbial composition, potentially influencing appetite regulation, energy metabolism, and obesity risk [17, 18].

Emerging evidence in pediatric populations suggests that low‑carbohydrate diets may improve specific metabolic risk factors such as insulin resistance [19]. Moderate carbohydrate restriction (approximately 35% of total energy intake) has been reported to be safe and potentially effective in improving insulin resistance and components of metabolic syndrome in adolescents [20, 21]. In contrast, modest reductions in carbohydrate intake (45–50% of total energy) without attention to carbohydrate quality have not demonstrated consistent benefits on insulin homeostasis or cardiovascular risk factors [22, 23]. Diets rich in fiber and complex carbohydrates may exert beneficial metabolic effects partly through modulation of the gut microbiota [24, 25]. To our knowledge, no previous randomized controlled trial has evaluated the effects of moderate carbohydrate restriction emphasizing high‑fiber sources on gut microbiota composition in children and adolescents with obesity. We hypothesized that modifying both the quantity and quality of dietary carbohydrates would improve body mass index (BMI), insulin resistance, other cardiometabolic risk factors, and gut microbiota composition. Therefore, the present randomized controlled trial aimed to evaluate the effects of a reduced‑carbohydrate diet (RCD) emphasizing high‑fiber sources compared with a standard diet (STD) on BMI as the primary outcome, as well as cardiometabolic risk factors and gut microbiota composition.

## Materials and methods

This 12-week, parallel-group randomized controlled trial was conducted from June 2022 to April 2023 to evaluate the effects of a reduced-carbohydrate diet (RCD) emphasizing high-fiber sources compared with a standard diet (STD) on cardiometabolic risk factors and gut microbiota composition in children and adolescents with obesity.

The study protocol was approved by the ethics committee and conducted in accordance with the Declaration of Helsinki. The study procedures were explained to both the children/adolescents and their parents or legal guardians. Written informed consent was obtained from the parents or legal guardians of all participants. In addition, oral assent was obtained from the children and adolescents after providing age-appropriate explanations of the study procedures. This approach was approved by the institutional Ethics Committee and is consistent with ethical guidelines for research involving minors. This manuscript was written in compliance with consolidated standards of reporting trials (CONSORT) statement.

Participants were recruited from the pediatric endocrinology clinic and referred to the nutrition and dietetics clinic at the National Nutrition and Food Technology Research Institute (NNFTRI). Inclusion criteria were age 7–16 years and a body mass index (BMI) z-score > 2.0 standard deviation scores (SDS) based on the World Health Organization (WHO) growth charts [26]. Exclusion criteria included: use of antibiotics (for more than three days within the last 3 months); use of corticosteroids, antipsychotics, or antidepressants; consumption of supplements affecting the gut microbiota (e.g., probiotics or prebiotics); participation in other weight loss programs; recent weight fluctuations; and a history of bariatric surgery. Additionally, children with obesity secondary to other medical conditions or those suffering from metabolic, renal, cardiovascular, thyroid, hepatic, or gastrointestinal diseases were excluded from the study.

The absolute body mass index (BMI) at the end of 12 weeks was designated as the primary outcome. Although BMI z-score was used for baseline eligibility and obesity classification, absolute BMI was selected as the primary outcome to optimize the sensitivity and interpretability of short-term treatment effects on body mass. In pediatric populations with obesity, BMI z-score may be less sensitive to change over a relatively short period (12 weeks), particularly at higher percentiles [27]. Nonetheless, changes in BMI z-score were also calculated and analyzed as a key secondary outcome.

Participants were randomly allocated to one of the two intervention groups using block randomization. The randomization sequence was generated using the Randomization.com website (http://www.randomization.com), creating six blocks of 14 participants each. The allocation list was prepared by the principal investigator and placed in sequentially numbered, sealed opaque envelopes prior to participant recruitment. Participants were enrolled by a trained dietitian at the nutrition clinic. After enrollment and completion of baseline assessments, the envelopes were opened sequentially to assign participants to the intervention groups, ensuring allocation concealment. Due to the nature of the dietary intervention, participants and research staff could not be blinded to group assignment. However, laboratory personnel and the data analyst were blinded to group allocation to minimize assessment bias.

Considering the desired difference of 2.5 kg/m^2^ in BMI between the two intervention groups [28, 29] and the standard deviation of 3.4 kg/m^2^ [30], with 80% power (β = 0.2), significance level 0.05 (α = 0.05), and accounting for 40% attrition, the required sample size was estimated to be 42 participants per group. The sample size calculation was performed using the formula for comparing two independent means [31].

At the first visit, the study protocol was fully explained to the adolescents and their parents. Written informed consent was obtained from parents or guardians, and oral assent was obtained from the adolescents. Participants received detailed instructions and an individualized meal plan (menu) based on their assigned group and estimated energy requirements. Diets were delivered face-to-face by a trained dietitian, and the intervention details were reported following the TIDieR checklist [32]. Participants in the RCD group received 30–40% of their energy from carbohydrates, 40–50% from fat, and 20% from protein, with emphasis on high-fiber sources including whole grains, legumes, nuts, seeds, vegetables, and fruits, while limiting added sugars to less than 5% of total energy intake. Participants in the STD group received 50–55% of their energy from carbohydrates, 25–30% from fat, and 15–20% from protein [33, 34]. These macronutrient distributions and the recommendation to limit added sugars represent the prescribed targets of the intervention. In contrast, the dietary intake values presented in the tables are derived from participants’ dietary recalls and therefore reflect actual intake during the study, which may differ from the prescribed targets because of variation in adherence and food choices.

Energy requirements for each participant were estimated based on height (kilojoules per centimeter) [35]. Both groups received an energy-restricted diet with a deficit of 250–500 kcal per day, tailored to individual weight management goals. Diet plans were provided as structured menus consisting of typical Iranian foods with detailed instructions (Supplementary Table 1). Participants were instructed to maintain their usual physical activity levels throughout the study to minimize confounding effects. To ensure adherence and provide support, a trained dietitian conducted weekly follow-up phone calls with all participants during the 12-week intervention.

Anthropometric measurements were performed at baseline and at the end of the study. Height was measured using a stadiometer (accuracy: 1 cm) and weight was recorded using a digital scale (accuracy: 100 g). Waist circumference was measured to the nearest 0.1 cm using a flexible tape at the midpoint between the lowest rib and the iliac crest, at the level of the umbilicus, without applying pressure to the body. Blood pressure was measured in a sitting position using a digital arm-type sphygmomanometer with an appropriate cuff size (Beurer, Germany); two readings were taken 30 s apart, and the average was recorded.

BMI-for-age and sex z-scores were calculated using WHO AnthroPlus software (based on the WHO Reference 2007 for children and adolescents aged 5–19 years). Obesity was defined as a BMI z-score > 2.0 standard deviations (SD) [26].

Body composition parameters, including body fat percentage (%) and skeletal muscle mass (kg), were determined using a multi-frequency bioelectrical impedance analyzer (BIA) (X-Contact 356; Jawon Medical Co., Seoul, South Korea) at the nutrition and dietetics clinic. To ensure the accuracy and reproducibility of BIA measurements, a standardized protocol was followed: participants were required to fast and refrain from fluid intake for at least 2 h prior to the assessment, empty their bladders, and rest in a seated position for 15 min before the test was performed.

To assess dietary intake and compliance with the assigned diet plans, three 24-h dietary recalls (comprising two weekdays and one weekend day) were collected at baseline, week 6, and week 12 (end of the intervention). Dietary data at baseline and week 12 were obtained through face-to-face interviews conducted by a trained dietitian at the clinic. Food portions were initially recorded using standard household measures and subsequently converted into grams [36]. Energy, carbohydrate, protein, total fat, fiber, saturated fat, and simple sugar intakes were analyzed using Nutritionist IV software (First Databank, San Bruno, CA, USA). All dietary recall interviews were conducted by a trained dietitian. Although 61 participants completed the intervention and were included in the primary outcome analyses, complete dietary recall data at 12 weeks were available for 51 participants. Therefore, dietary intake analyses were conducted based on participants with complete dietary records at that time point.

Physical activity levels were assessed at baseline and at the end of the intervention using the Modifiable Activity Questionnaire (MAQ), which has been previously validated [37]. Participants reported leisure-time physical activities they had engaged in at least ten times during the preceding three months. For each identified activity, the frequency and duration were recorded. The intensity of each activity was assigned a Metabolic Equivalent of Task (MET) value, and the results were expressed as MET-hours per week.

Blood samples were collected after a 12-h overnight fast at baseline and at the end of the intervention. Samples were centrifuged at 3,000 × g for 10 min to separate the plasma, which was then aliquoted and stored at − 80 °C until analysis.

Plasma glucose, total cholesterol, triglycerides (TG), high-density lipoprotein cholesterol (HDL-C), aspartate aminotransferase (AST), alanine transaminase (ALT) and gamma-glutamyl transferase (GGT) have been measured by Pictus 700 Clinical chemistry analyzer, Diatron MI Plc (Budapest, Hungary) and enzymatic methods (AUDIT, Delta Darman Part, Tehran, Iran). The insulin concentration was measured using Biotek Epoch microplate reader (BioTek Instruments, Inc., Highland Park, USA) and Enzyme-linked immunosorbent assay method (Monobind insulin microplate ELISA test, Monobind Inc., USA).

Intra- and inter-assay coefficients of variation (CVs) were < 2% for glucose, TG, HDL-C, cholesterol, GGT and < 4.0% for insulin, ALT and AST. Low-density lipoprotein cholesterol (LDL-C) in plasma samples with TG levels equal to or less than 400 mg/dL was calculated using the Friedewald formula as follows: LDL-C = Total cholesterol – (HDL-C + TG/5) [38]. Insulin resistance was estimated using the homeostatic model assessment of insulin resistance (HOMA-IR), which uses the following formula: HOMA-IR = [fasting insulin (µU/ml) × fasting plasma glucose (mg/dl)]/405 [39].

To investigate the intestinal microbiota, stool samples of the participants were used. Stool samples were collected in sterile cups at baseline and end of the intervention. After collection, the samples were stored at −80 °C. DNA stool kits with standard protocols were used for DNA extraction. To investigate the intestinal microbiota, all microbial DNA was extracted from the stool sample using the QIAamp DNA stool mini kit (Qiagen, Hilden, Germany). To determine the purity of extracted DNA, the absorption ratio of A260/A280 was measured using a spectrophotometer and a Nanodrop device to determine the concentration of extracted DNA (NanoDrop, Thermo Scientific).

Specific primers were targeted the bacterial 16S rRNA genes to determine the abundance of bacterial phyla, including Actinobacteria, Firmicutes, Bacteroidetes, Proteobacteria and Verrucomicrobia and genera, including *Prevotella, Lactobacillus, Bifidobacterium, Bacteroides, Escherichia.* Sequences of specific primers used in this study are shown in Supplementary Table 2. Bacterial abundance of fecal samples was Analyzed using quantitative real-time PCR based on SYBER Green method (48-well StepOne™ real-time PCR thermal cycler, Applied Biosystems, Foster City, CA, USA). Each quantitative real-time PCR reaction included SYBR green master mix (Yekta Tajhiz Azma, Iran), each of the specific 16 s rRNA primers, and the DNA template was performed in triplicate using LightCycler® 8-Tube Strips. The amplification program was designed regarding the appropriate annealing temperature: an initial denaturation step 1 cycle of 95 °C for 60 s, followed by 40 cycles of denaturation at 95 °C for 5 s, annealing at 55 °C for 30 s, and extension at 72 °C for 30 s. Melting curve analysis was carried out after amplification to control the specificity of PCR reaction, followed by one cycle at 95 °C for 5 s, 60 °C for 60 s, and 95 °C for 1s. Calculation of the DNA concentration of each bacterium from fecal samples was conducted by standard curves.

Statistical analyses were performed using SPSS software version 26 (SPSS Inc., Chicago, IL, USA). The normality of variables was assessed using the Kolmogorov–Smirnov test. BMI was considered the primary outcome. Categorical variables were compared between intervention groups using the chi-square test. Continuous variables are presented as mean ± standard deviation (SD). Within-group changes from baseline to week 12 were assessed using paired Student’s t-tests. The primary endpoint was defined as the change from baseline to week 12; therefore, within-group analyses focused on baseline-to-week-12 changes. The week-6 assessment was included mainly to monitor interim trends and dietary adherence. For variables that were not normally distributed, non-parametric tests were applied. The Mann–Whitney U test was used for between-group comparisons and the Wilcoxon signed-rank test for within-group comparisons. Between-group differences at week 12 were evaluated using analysis of covariance (ANCOVA), with baseline values included as covariates. Adjusted mean differences between groups with corresponding 95% confidence intervals (95% CI) were calculated. Effect sizes were reported as partial eta squared (ηp^2^) derived from the ANCOVA models. Effect sizes were interpreted using partial eta squared (ηp2), with values of approximately 0.01, 0.06, and 0.14 representing small, medium, and large effects, respectively, according to Cohen’s guidelines [40]. A *p*-value < 0.05 was considered statistically significant. Statistical analyses were conducted using a per-protocol approach, including only participants who completed the study and had complete outcome data. No intention-to-treat (ITT) analysis or imputation for missing data was performed because only the pre-specified primary analyses registered in the study protocol were conducted.

## Results

A total of 182 children and adolescents were screened for eligibility. Of these, 98 individuals were excluded for the following reasons: (1) BMI-for-age z-score < 2.0 (*n* = 36); (2) use of medications including antibiotics, psychiatric drugs, or hormone therapy (*n* = 34); (3) inability to attend scheduled study visits (*n* = 5); (4) declining to participate (*n* = 10); and (5) other miscellaneous reasons (*n* = 13).

**Fig. 1:**
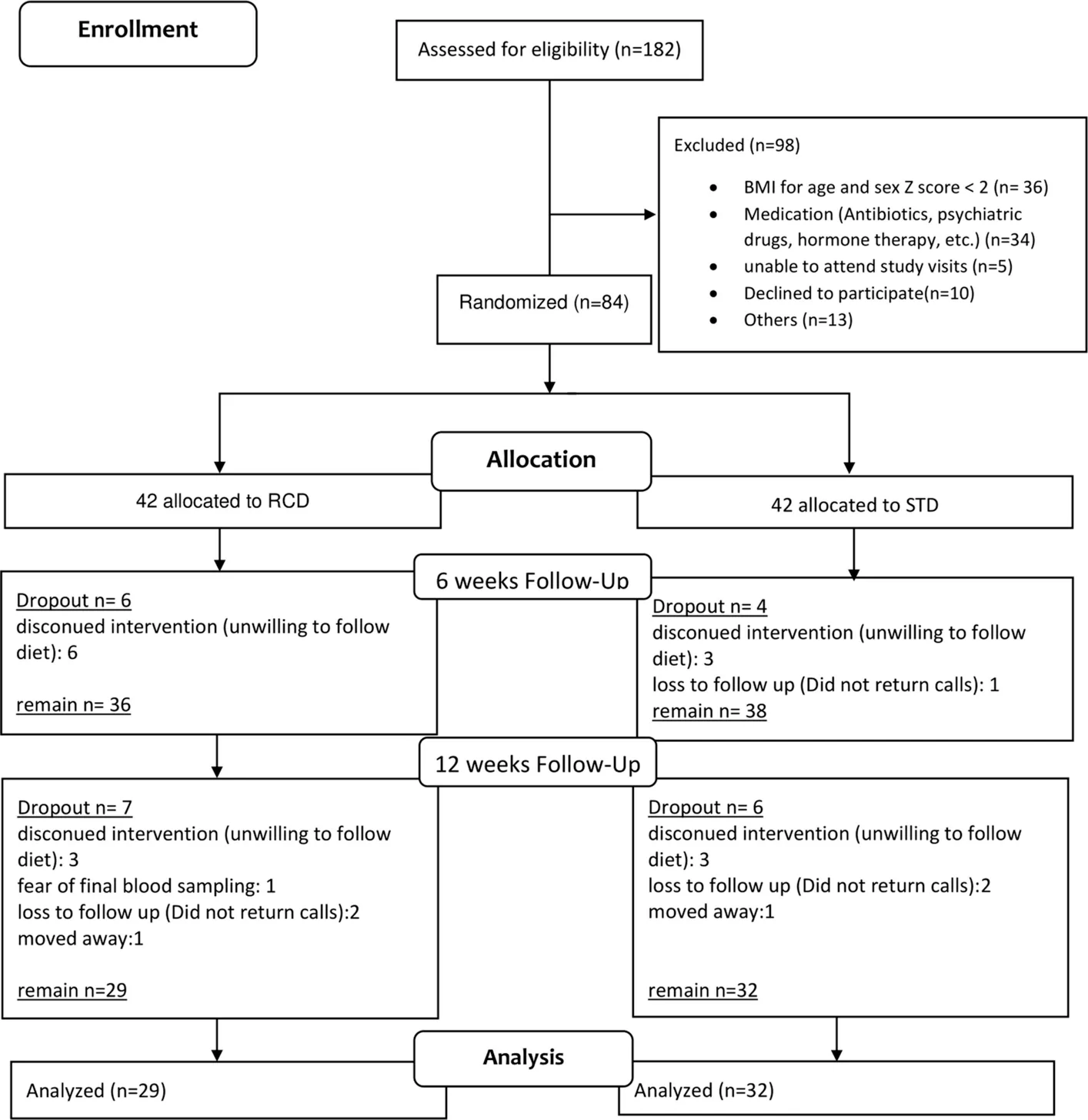
The CONSORT flow diagram of study participants

The remaining 84 participants were randomly assigned to either the reduced-carbohydrate diet (RCD; *n* = 42) or the standard diet (STD; *n* = 42) group. The flow of participants through the study, from initial screening to final analysis, is presented in Fig. 1. At the end of the 12-week intervention, 29 (69.0%) participants in the RCD group and 32 (76.1%) in the STD group completed the study. No adverse events related to the dietary intervention were reported during the study period. To assess potential attrition bias, baseline characteristics including age, sex, anthropometric indices, and physical activity levels were compared between participants who completed the study and those who withdrew. No significant differences were observed (all *p* > 0.05) (Supplementary Table 3).

Baseline characteristics of the participants are presented in Table 1. The mean (SD) age of the 84 participants (54 boys and 30 girls) was 11.5 ± 2.2 years, and the mean BMI z-score was 2.60 ± 0.60. After 12 weeks of intervention, BMI, BMI z-score, waist circumference, and percent body fat were significantly reduced in both groups (Table 2). Body weight decreased significantly in the STD group (*p* = 0.023), whereas the reduction in the RCD group did not reach statistical significance (*p* = 0.075). Skeletal muscle mass did not change significantly in either group.

**Table 1:** Baseline characteristics of study participants by diet group

	RCD (*n* = 42)	STD (*n* = 42)
Age, years	11.4 ± 1.8	11.5 ± 2.7
Female sex	13 (31.0)	17 (40.5)
Height, cm	148.3 ± 11.1	152.9 ± 13.1
height, z score	0.37 ± 0.99	0.81 ± 1.06
weight, kg	59.6 ± 12.8	62.1 ± 15.0
BMI, kg/m^2^	26.8 ± 2.8	26.1 ± 3.1
BMI, z score, SD units	2.66 ± 0.51	2.59 ± 0.68
Waist circumference, cm	88.0 ± 10.2	88.9 ± 9.8
Percent of body fat	28.9 ± 5.4	29.0 ± 5.5
Skeletal muscle mass, kg	23.0 ± 4.2	24.5 ± 5.1
Glucose, mg/dL	88.0 ± 12.4	85.98 ± 8.9
Insulin, µIU/mL	17.6 ± 9.5	16.7 ± 8.6
HOMA-IR	4.1 ± 2.7	3.6 ± 2.0
Cholesterol, mg/dl	164.2 ± 33.7	170.9 ± 37.3
HDL-C, mg/dL	43.9 ± 6.6	42.5 ± 6.4
LDL-C, mg/dL	92.0 ± 27.3	103.2 ± 31.0
TG, mg/dL	141.3 ± 69.6	125.7 ± 59.9
TG/HDL-C	3.3 ± 1.7	3.0 ± 1.6
GGT, U/L	18.6 ± 5.2	16.6 ± 4.5
AST, U/L	30.8 ± 10.6	28.7 ± 7.9
ALT, U/L	28.3 ± 12.3	27.3 ± 18.3
Systolic blood pressure, mmHg	107.94 ± 10.71	111.75 ± 8.45
Diastolic blood pressure, mmHg	69.21 ± 10.50	70.87 ± 7.68
Physical activity (MET/h/week)	7.9 ± 13.0	4.9 ± 9.2
More than 6 h of screen time, n (%)	35 (83.3)	28 (66.7)
Dietary intakes		
Energy (Kcal/day)	1718 ± 579	1824 ± 544
Carbohydrate (% E)	49.9 ± 9.6	52.3 ± 8.7
Protein (% E)	16.7 ± 7.8	14.4 ± 3.4
Fat (% E)	34.5 ± 8.8	34.9 ± 8.9
Saturated fatty acid (% E)	12.1 ± 5.3	12.9 ± 6.6
Sugar (% E)	15.1 ± 6.4	15.6 ± 6.7
Fiber (gr)	7.6 ± 3.7	9.4 ± 4.2
Fiber (g/1000 kcal)	4.6 ± 2.0	5.4 ± 2.5

Continuous variables are presented as mean ± standard deviation; categorical variables are presented as frequency (percent). Between-group comparisons were performed using independent Student’s t-test or Mann–Whitney U test, as appropriate. Categorical variables were compared using the chi-square test

*Abbreviations*: *RCD* Restricted carbohydrate diet, *STD* Standard diet, *BMI* Body mass index, *HOMA-IR* Homeostatic model assessment of insulin resistance, *HDL* High density lipoprotein cholesterol, *LDL* Low density lipoprotein cholesterol, *TG* Triglycerides, *GGT* Gamma-glutamyl transferase, *AST* Aspartate aminotransferase, *ALT* Alanine aminotransferase

**Table 2:** Changes of anthropometric indices, biochemical outcomes and blood pressure at the end of intervention by diet group

Outcome	Group	Time	Mean (SD)	Within-group Mean Difference (95% CI)	*p*-value	Between-group Mean Difference (95% CI)	*p*-value	Effect size
Weight, kg	RCD	Baseline	57.7 ± 12.2					
		Week 12	56.4 ± 11.1	−1.27 (−2.68, 0.14)	0.075			
	STD	Baseline	64.4 ± 19.1					
		Week 12	63.1 ± 18.5	−1.25 (−2.31, −0.19)	**0.023**	−0.46 (−2.13, 1.206)	0.582	0.01
BMI, kg/m^2^	RCD	Baseline	26.3 ± 2.8					
		Week 12	25.2 ± 3.1	−1.12 (−1.72, −0.52)	**0.001**			
	STD	Baseline	26.7 ± 4.2					
		Week 12	25.5 ± 4.1	−1.17 (−1.53, −0.81)	**0.000**	0.903 (−0.28, 2.08)	0.588	0.04
BMI z-score	RCD	Baseline	2.6 ± 0.5					
		Week 12	2.3 ± 0.6	−0.30 (−0.41, −0.19)	**0.000**			
	STD	Baseline	2.5 ± 0.6					
		Week 12	2.2 ± 0.6	−0.31 (−0.39, −0.24)	**0.000**	0.18 (−0.12, 0.49)	0.236	0.02
PBF, %	RCD	Baseline	28.4 ± 5.7					
		Week 12	27.6 ± 5.1	−2.30 (−4.51, −0.10)	**0.041**			
	STD	Baseline	28.4 ± 5.4					
		Week 12	26.6 ± 6.0	−1.77 (−2.90, −0.63)	**0.003**	0.75 (−1.89, 3.39)	0.572	0.01
WC, cm	RCD	Baseline	87.0 ± 7.5					
		Week 12	84.1 ± 7.8	−2.93 (−4.28, −1.58)	**0.000**			
	STD	Baseline	90.0 ± 10.7					
		Week 12	87.1 ± 10.2	−2.88 (−4.11, −1.64)	**0.000**	0.97 (−1.48, 3.43)	0.429	0.01
SMM, kg	RCD	Baseline	22.6 ± 3.8					
		Week 12	23.0 ± 3.5	0.42 (−0.38, 1.22)	0.292			
	STD	Baseline	25.1 ± 5.3					
		Week 12	25.3 ± 5.2	0.19 (−0.18, 0.55)	0.305	−0.54 (−1.77, 0.69)	0.380	0.01
Glucose, mg/dL	RCD	Baseline	89.8 ± 14.6					
		Week 12	88.6 ± 13.5	−1.24 (−4.59, 2.11)	0.454			
	STD	Baseline	85.2 ± 9.3					
		Week 12	85.3 ± 9.5	0.06 (−2.52, 2.65)	0.960	4.11 (−1.93, 10.16)	0.178	0.03
Insulin, µIU/mL	RCD	Baseline	16.7 ± 10.1					
		Week 12	14.3 ± 6.7	−2.42 (−5.69, 0.85)	0.141			
	STD	Baseline	16.4 ± 9.5					
		Week 12	14.0 ± 5.8	−2.43 (−5.35, 0.48)	0.098	1.05 (−2.11, 4.20)	0.509	0.01
HOMA-IR	RCD	Baseline	3.9 ± 3.0					
		Week 12	3.2 ± 1.7	−0.74 (−1.62, 0.15)	0.099			
	STD	Baseline	3.5 ± 2.3					
		Week 12	3.0 ± 1.3	−0.55 (−1.25, 0.16)	0.123	0.43 (−035, 1.20)	0.273	0.02
ALT, U/L	RCD	Baseline	29.2 ± 13.8					
		Week 12	21.9 ± 6.1	−7.29 (−12.46, −2.11)	**0.008**			
	STD	Baseline	25.8 ± 17.1					
		Week 12	20.0 ± 7.5	−5.77 (−9.99, −1.56)	**0.009**	2.08 (−1.62, 5.78)	0.264	0.02
AST, U/L	RCD	Baseline	32.7 ± 8.4					
		Week 12	30.5 ± 7.1	−2.21 (−5.98, 1.55)	0.238			
	STD	Baseline	28.7 ± 6.1					
		Week 12	26.3 ± 4.4	−2.39 (−4.12, −0.65)	**0.009**	3.35 (0.37, 6.32)	**0.028**	0.03
GGT, U/L	RCD	Baseline	18.6 ± 5.8					
		Week 12	17.3 ± 6.7	−1.30 (−3.57, 0.98)	0.252			
	STD	Baseline	16.9 ± 3.9					
		Week 12	15.7 ± 4.4	−1.13 (−2.28, 0.02)	0.054	1.81 (−1.21, 4.84)	0.235	0.03
Cholesterol, mg/dl	RCD	Baseline	164.2 ± 36.8					
		Week 12	155.5 ± 38.6	−8.72 (−21.03, 3.58)	0.158			
	STD	Baseline	172.3 ± 39.8					
		Week 12	169.7 ± 36.2	−2.58 (−10.72, 5.56)	0.522	−15.68 (−35.46, 4.10)	0.118	0.04
HDL-C, mg/dL	RCD	Baseline	43.3 ± 6.8					
		Week 12	42.8 ± 6.6	−0.48 (−3.19, 2.23)	0.718			
	STD	Baseline	41.1 ± 5.4					
		Week 12	41.1 ± 5.4	0.00 (−1.36, 1.36)	1.000	1.31 (−1.83, 4.45)	0.408	0.01
LDL-C, mg/dL	RCD	Baseline	94.2 ± 28.4					
		Week 12	93.7 ± 32.8	−0.46 (−10.23, 9.32)	0.925			
	STD	Baseline	105. 2 ± 33.5					
		Week 12	101.5 ± 30.1	−3.65 (−11.43, 4.12)	0.345	−9.46 (−26.01, 7.10)	0.258	0.02
TG, mg/dL	RCD	Baseline	133.8 ± 76.5					
		Week 12	98.7 ± 42.7	−43.62 (−67.2, −20.0)	**0.001**			
	STD	Baseline	130.0 ± 62.6					
		Week 12	135.3 ± 81.9	5.35 (−19.44, 30.15)	0.662	−40.31 (−73.79, −6.82)	**0.019**	0.09
TG/HDL-C	RCD	Baseline	3.2 ± 1.9					
		Week 12	2.4 ± 1.0	−0.84 (−1.52, −0.16)	**0.018**			
	STD	Baseline	3.2 ± 1.7					
		Week 12	3.3 ± 2.1	0.11 (−0.45, 0.68)	0.684	−1.03 (−1.85, −0.20)	**0.016**	0.098
Systolic blood pressure, mmHg	RCD	Baseline	107.6 ± 9.8					
		Week 12	103.9 ± 8.4	−3.67 (−8.52, 1.19)	0.127			
	STD	Baseline	109.7 ± 9.6					
		Week 12	105.9 ± 13.4	−3.75 (−13.39, 5.89)	0.410	−1.85 (−9.20, 5.50)	0.612	0.00
Diastolic blood pressure, mmHg	RCD	Baseline	68.6 ± 11.1					
		Week 12	65.9 ± 9.5	−2.67 (−10.29, 4.95)	0.465			
	STD	Baseline	71.0 ± 7.7					
		Week 12	70.4 ± 11.3	−0.64 (−9.86, 8.59)	0.881	−5.288 (−11.67, 1.09)	0.101	0.08

Continuous variables are presented as mean ± SD. Within-group comparisons (baseline vs. week 12) were performed using paired Student’s t-test. Between-group differences in changes from baseline to week 12 were analyzed using analysis of covariance (ANCOVA), adjusting for baseline values. The number of participants analyzed in each group was *n* = 29 for the RCD group and n = 32 for the STD group

Bold values indicate statistically significant results (*p* < 0.05)

*Abbreviations*: *RCD*, Restricted carbohydrate diet, *STD* Standard diet, *BMI* Body mass index, *PBF* percent body fat, *WC* Waist circumference, *SMM* Skeletal muscle mass, *HOMA-IR* Homeostatic model assessment of insulin resistance, *HDL-C* High-density lipoprotein cholesterol, *LDL-C* Low-density lipoprotein cholesterol, *TG* Triglycerides, *GGT* Gamma-glutamyl transferase, *AST* Aspartate aminotransferase, *ALT* Alanine aminotransferase

Biochemical outcomes are presented in Table 2. Triglyceride (TG) concentrations decreased significantly in the RCD group (− 43.62 mg/dL, 95% CI − 67.20 to − 20.00; *p* = 0.001), whereas no significant change was observed in the STD group (5.35 mg/dL, 95% CI − 19.44 to 30.15; *p* = 0.662). Accordingly, the reduction in TG was significantly greater in the RCD group compared with the STD group (between‑group mean difference − 40.31 mg/dL, 95% CI − 73.79 to − 6.82; *p* = 0.019). Similarly, the TG/HDL‑C ratio decreased significantly in the RCD group (− 0.84, 95% CI − 1.52 to − 0.16; *p* = 0.018) but not in the STD group (0.11, 95% CI − 0.45 to 0.68; *p* = 0.684), resulting in a significant between‑group difference (− 1.03, 95% CI − 1.85 to − 0.20; *p* = 0.016).

ALT levels decreased significantly in both groups (RCD: − 7.29 U/L, 95% CI − 12.46 to − 2.11; p = 0.008; STD: − 5.77 U/L, 95% CI − 9.99 to − 1.56; *p* = 0.009). AST levels declined significantly in the STD group (− 2.39 U/L, 95% CI − 4.12 to − 0.65; *p* = 0.009), whereas the reduction in the RCD group was not statistically significant (− 2.21 U/L, 95% CI − 5.98 to 1.55; *p* = 0.238). At week 12, AST levels were significantly lower in the STD group compared with the RCD group (26.3 ± 4.4 vs. 30.5 ± 7.1 U/L; *p* = 0.028). No significant changes or between‑group differences were observed for fasting glucose, insulin, total cholesterol, HDL‑C, LDL‑C, HOMA‑IR, GGT, or systolic and diastolic blood pressure. Effect size analysis indicated that triglycerides and the TG/HDL-C ratio showed the largest between-group effects, while effect sizes for the other outcomes were small (Table 2).

**Fig. 2:**
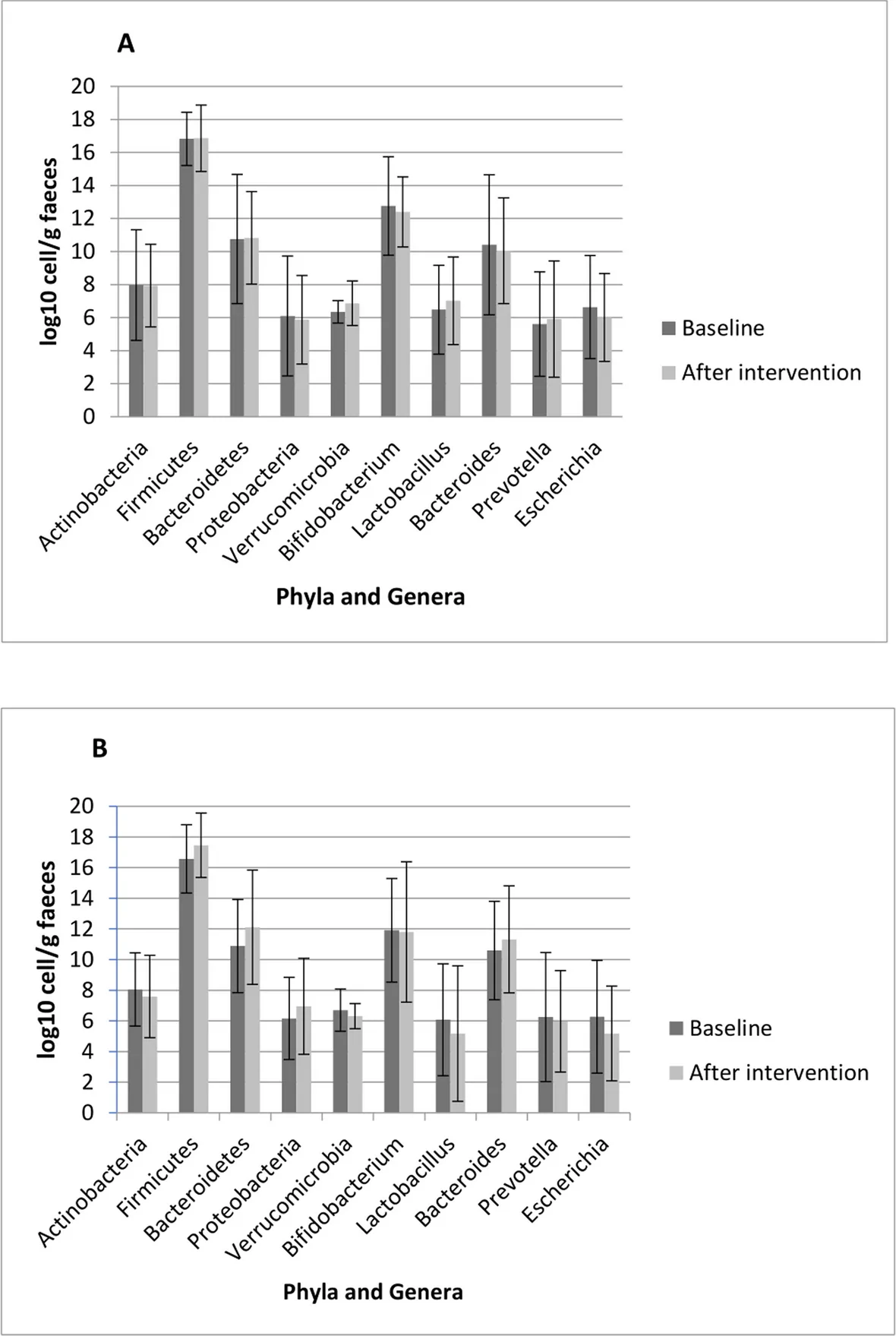
Bacterial abundance at the phylum and genus level before and after the intervention. **A** Bacterial load of fecal samples before and after reduced carbohydrate diet (*N* = 22), (**B**) Bacterial load of fecal samples before and after standard diet (*N* = 23)

No significant changes within groups or differences between groups were observed in the abundance of investigated bacteria at the phylum and genus levels (*p* > 0.05) (Fig. 2). However, the frequency of Verrucomicrobia after the intervention increased in RCD group and decreased in STD group (mean of changes: 0.52 ± 1.34 vs. −0.39 ± 1.34 log10 cell/gram of faeces); these changes showed a significant difference between groups. Differences in the changes in the abundance of Verrucomicrobia showed medium effect size (Supplementary Table 4).

As shown in Table 3, total energy intake decreased significantly in both groups by week 12 (RCD: − 385 kcal/day, *p* = 0.008; STD: − 534 kcal/day, *p* = 0.001), with no significant between‑group difference (*p* = 0.538). At week 12, the percentage of energy from carbohydrates was significantly lower in the RCD group than in the STD group (46.9 ± 9.6 vs. 56.1 ± 7.7; between‑group *p* = 0.003), and sugar intake also decreased more in the RCD group (− 4.17%, *p* = 0.037) compared with the STD group (between‑group *p* = 0.026). Fat intake was significantly higher in the RCD group compared with the STD group at week 12 (between‑group *p* = 0.042). Protein intake increased significantly within both groups (both *p* < 0.05), with no between‑group difference, and no significant differences were observed for saturated fat or fiber intake (all *p* > 0.05).

**Table 3:** Dietary intake of participants during the intervention

Outcome	Group	Time	Mean ± SD	Within-group Mean Difference (95% CI)	***p***-value	Between-group Mean Difference (95% CI)	***p***-value	Effect size
Energy (Kcal/day)	RCD	Baseline	1625 ± 449					
		Week 6	1589 ± 211					
		Week 12	1239 ± 386	−385 (−654, −116)	**0.008**			
	STD	Baseline	1880 ± 556					
		Week 6	1641 ± 262*					
		Week 12	1346 ± 450	−534 (−812, −256)	**0.001**	−83.95 (−357.26, 189.36)	0.538	0.01
Carbohydrate (% of Energy)	RCD	Baseline	51.3 ± 6.5					
		Week 6	40.4 ± 7.03*					
		Week 12	46.9 ± 9.6	−3.57 (−9.16,2.03)	0.196			
	STD	Baseline	53.4 ± 7.1					
		Week 6	41.7 ± 5.7*					
		Week 12	56.1 ± 7.7	2.66 (−1.72, 7.06)	0.222	−8.84 (−13.81, −3.02)	**0.003**	0.19
Protein (% of Energy)	RCD	Baseline	15.6 ± 2.4					
		Week 6	20.7 ± 3.8*					
		Week 12	19.1 ± 5.9	3.44 (0.84, 6.03)	**0.012**			
	STD	Baseline	14.3 ± 2.6					
		Week 6	19.2 ± 3.7*					
		Week 12	18.1 ± 4.6	3.76 (1.76, 5.76)	**0.001**	0.24 (−2.96, 3.45)	0.880	0.01
Fat (% of Energy)	RCD	Baseline	34.5 ± 6.4					
		Week 6	40.2 ± 8.6*					
		Week 12	34.7 ± 7.0	0.22 (−3.39, 3.84)	0.897			
	STD	Baseline	33.9 ± 8.6					
		Week 6	39.9 ± 5.2*					
		Week 12	29.1 ± 9.5	−4.79 (−10.19, 0.62)	0.080	5.59 (0.12, 10.99)	**0.042**	0.10
Saturated fatty acid (% of Energy)	RCD	Baseline	12.3 ± 3.2					
		Week 6	11.2 ± 2.4					
		Week 12	11.5 ± 3.4	−0.80 (−1.91, 0.32)	0.149			
	STD	Baseline	11.8 ± 6.3					
		Week 6	10.48 ± 2.3					
		Week 12	9.6 ± 3.5	−2.18 (−4.80, 0.45)	0.100	1.74 (−0.29, 3.77)	0.091	0.068
Sugar (% of Energy)	RCD	Baseline	15.8 ± 7.0					
		Week 6	13.7 ± 5.1					
		Week 12	11.6 ± 2.8	−4.17 (−8.05, −0.28)	**0.037**			
	STD	Baseline	14.3 ± 5.0					
		Week 6	11.7 ± 4.6*					
		Week 12	15.1 ± 5.4	0.80 (−2.53, 4.13)	0.625	−3.22 (−6.04, −0.40)	**0.026**	0.12
Fiber (gr)	RCD	Baseline	7.7 ± 4.0					
		Week 6	11.3 ± 3.3*					
		Week 12	7.4 ± 3.2	−0.33 (−2.64, 1.98)	0.766			
	STD	Baseline	9.9 ± 4.4					
		Week 6	9.9 ± 3.9					
		Week 12	8.9 ± 3.2	−0.98 (−3.05, 1.08)	0.335	−1.31 (−3.36, 0.74)	0.21	0.04
Fiber (g/1000 kcal)	RCD	Baseline	4.7 ± 2.3					
		Week 6	7.1 ± 1.8*					
		Week 12	6.1 ± 2.5	1.33 (−0.15, 2.82)	0.076			
	STD	Baseline	5.5 ± 2.3					
		Week 6	6.2 ± 2.6					
		Week 12	7.4 ± 3.7	1.87 (0.35, 3.38)	**0.018**	−1.01 (−3.01, 0.99)	0.317	0.03

Within-group comparisons (baseline vs. week 12) were performed using paired Student’s t-test or Wilcoxon signed-rank test, as appropriate. Between-group differences in changes from baseline to week 12 were analyzed using analysis of covariance (ANCOVA), adjusting for baseline values

Bold values indicate statistically significant results (*p* < 0.05)

Values at week 6 and week 12 are presented as mean ± SD

Dietary intake values were calculated based on participants with complete 24-h dietary recall data at each time point (Week 6: *n* = 57; RCD, *n* = 25; STD, *n* = 32; Week 12: *n* = 51; RCD, *n* = 24; STD, *n* = 27)

*Abbreviations*: *RCD* Restricted carbohydrate diet, *STD* Standard diet

^*^p < 0.05 for within-group comparisons (baseline vs. week 6)

## Discussion

In this randomized controlled trial, we examined the effects of a reduced-carbohydrate diet (RCD) emphasizing high-fiber carbohydrate sources compared with a standard diet (STD) on BMI, cardiometabolic indicators, and gut microbiota composition over a 12-week period. Both dietary interventions resulted in reductions in nearly all anthropometric indices. In addition, a more pronounced decrease in TG levels and the TG/HDL-C ratio was observed in the RCD group. Regarding gut microbiota, the abundance of Verrucomicrobia increased in the RCD group but decreased in the STD group, while no other significant changes were detected in the abundance of the assessed bacterial phyla or genera.

We did not observe any superiority of moderate carbohydrate restriction compared with the standard diet in reducing BMI, BMI z‑score, body fat percentage, or waist circumference during the 12‑week intervention. Our dietary intervention was as effective as other behavioral change programs [41], although the follow‑up durations in previous studies varied. Consistent with the findings of Casazza et al., our results showed that a reduced‑carbohydrate diet (providing 42% of energy from carbohydrates) and a standard diet produced similarly favorable effects on weight and body fat, with no significant differences in weight loss between the groups [20]. Similarly, other studies have reported improvements in weight status and body composition among children and adolescents with obesity in both low‑fat diets and reduced‑carbohydrate diets (35–50% of energy from carbohydrates) [22, 42]. Overall, regarding anthropometric outcomes, it appears that calorie restriction regardless of the macronutrient composition of the diet can beneficially reduce weight and adiposity‑related risk factors.

Regarding biochemical indices, neither group showed significant changes in fasting glucose or insulin levels at the end of the intervention. These findings contrast with previous studies that reported improvements in insulin resistance among children and adolescents following three months of hypocaloric or low-carbohydrate diets. For instance, Demol et al. observed significant reductions in insulin and HOMA-IR levels only in groups where carbohydrate intake was limited to 20% of total energy whereas in our study the RCD provided approximately 42% of total energy from carbohydrates [43]. Other studies have shown comparable HOMA-IR improvements across various dietary patterns, including hypocaloric, very-low-carbohydrate, low-fat, or modified-carbohydrate diets [42, 44]. It is possible that the effect of diet on fasting insulin levels is mediated more by calorie restriction and subsequent BMI changes rather than by macronutrient composition itself [45]. Furthermore, since postprandial insulin is considered a more sensitive early biomarker for detecting prediabetes compared to fasting insulin [46], its measurement should be considered in future studies.

A greater reduction in TG levels was observed in the RCD group compared with the STD group. This finding is consistent with studies evaluating very-low-carbohydrate diets among adolescents [43, 47]. For example, Kirk et al. reported a significant decrease in TG levels only in the low-carbohydrate diet group over a 3-month period [47]. Moreover, meta-analyses in adults examining low-carbohydrate diets and cardiovascular risk factors have suggested metabolic advantages of carbohydrate restriction, including reductions in TG levels [48, 49]. In line with our findings, Casazza et al. demonstrated that even moderate carbohydrate restriction (42% of energy from carbohydrates) could significantly reduce TG levels [20]. Moderate carbohydrate restriction may improve triglyceride concentrations through several well-described metabolic pathways. Lower carbohydrate intake reduces hepatic de novo lipogenesis, leading to decreased production and secretion of very-low-density lipoprotein (VLDL) particles, the primary carriers of circulating triglycerides. Additionally, reduced postprandial insulin secretion may decrease hepatic triglyceride synthesis and improve lipid handling. Together, these mechanisms provide a physiological explanation for the more pronounced reduction in TG observed in the RCD group [50]. Consistent with the reduction in TG levels, the TG/HDL-C ratio also decreased significantly in the RCD group. The TG/HDL-C ratio has been proposed as a sensitive predictor and risk marker for insulin resistance, metabolic syndrome, and cardiovascular disease in children and adolescents [51, 52]. Although we did not observe significant changes in HOMA-IR, the TG/HDL-C ratio considered a surrogate marker of insulin resistance [51], improved significantly in the RCD group compared with the STD group. Given the similar weight loss and reported calorie intake in both groups, these findings suggest that macronutrient composition, independent of total energy intake, may contribute to improvements in TG levels. This effect may be explained by the role of carbohydrates particularly simple sugars in stimulating insulin secretion and promoting triglyceride-rich lipoprotein synthesis. Accordingly, carbohydrate restriction, especially limiting simple sugars, is commonly recommended in the nutritional management of hypertriglyceridemia in children and adolescents, and in severe cases, complete elimination of simple sugars may be advised [53, 54].

Among the investigated bacterial taxa, only changes in the abundance of Verrucomicrobia differed significantly between the two intervention groups. To the best of our knowledge, this study is the first to suggest that moderate carbohydrate restriction may have modest effects on Verrucomicrobia abundance in adolescents. However, given the relatively small sample size and the use of targeted qPCR rather than comprehensive sequencing approaches, these microbiota findings should be considered exploratory and interpreted with caution.

The only known representative of this phylum in the human gut is *Akkermansia muciniphila*, which has recently been proposed as a hallmark of a healthy gut because of its favorable metabolic effects, despite its relatively low abundance in the gut microbiota. Previous studies have reported a strong association between Verrucomicrobia abundance and pediatric obesity [55].

In addition, a systematic review suggested that *Akkermansia muciniphila* may be a potential target for dietary interventions aimed at improving metabolic status [56]. Changes in gut microbiota may also contribute to the metabolic effects of moderate carbohydrate restriction. Akkermansia muciniphila, as the main representative of the Verrucomicrobia phylum, is known to degrade mucin and enhance intestinal barrier function, which has been linked to improved metabolic health, reduced inflammation, and better lipid metabolism. Therefore, an increased abundance of Akkermansia may partly explain the favorable changes in TG metabolism observed in the RCD group. Although the magnitude of change was modest, these findings are consistent with proposed mechanisms linking diet composition, gut barrier integrity, and systemic metabolic outcomes [57–60]. No other significant changes in gut microbiota composition were observed in the present study.

A possible explanation is that fiber intake did not reach the intended level of approximately 14 g per 1000 kacl, despite the observed increase in fiber consumption in both groups. Zhang et al. reported that a high-fiber formula-based diet providing 50 g of fiber per day could significantly alter gut microbiota composition in children [25]. Differences in the changes in the abundance of Verrucomicrobia showed medium effect size. Nevertheless, because the microbiota assessment was based on targeted qPCR and involved a relatively small sample size, these findings should be considered preliminary and require confirmation in future studies using larger cohorts and comprehensive microbiome profiling methods such as 16S rRNA sequencing or metagenomic analysis.

No adverse effects related to the dietary interventions were reported during the study. This contrasts with findings from studies of very low‑carbohydrate diets, in which symptoms such as weakness, headache, diarrhea, constipation, and nausea have been reported [43]. Very low‑carbohydrate diets (10–25% of total energy intake from carbohydrates) have also been associated with impaired growth in children, and such highly restrictive dietary patterns may be difficult for pediatric populations to sustain [47, 61]. In contrast, moderate carbohydrate restriction (< 40% of total energy intake) can achieve nutritional adequacy when diet plans emphasize nutrient‑dense foods [62].

However, adherence to dietary prescriptions in pediatric populations can be challenging. In our study, although the intervention emphasized high‑fiber carbohydrate sources, fiber intake did not significantly differ between groups at the end of the intervention. In addition, the actual carbohydrate intake in the reduced‑carbohydrate group at 12 weeks (46% of total energy intake) was higher than the intended target range (30–40%). This suggests that the dietary contrast between groups was only partially achieved, which may have attenuated the magnitude of the observed intervention effects.

Despite the flexibility of our dietary intervention and the support provided through weekly follow-up calls, the dropout rate was 27.3%. This is consistent with previous pediatric weight loss programs, which have reported high attrition rates ranging from 27 to 73% [63]. Importantly, the baseline characteristics of the participants who completed the study did not differ significantly from those who dropped out, which helps mitigate the risk of attrition bias.

The strengths of the present study include the implementation of an individualized dietary intervention tailored to participants’ habitual dietary intakes, as well as close and structured follow-up throughout the intervention period. This study has several limitations. First, as in many nutritional interventions, the sample size was relatively small and assessment of dietary adherence was challenging. Participants were not provided with meals or food items during the intervention; therefore, adherence depended entirely on participants’ and families’ food choices. Although this approach reflects a real-world setting, it may have introduced additional variability in compliance with the prescribed macronutrient targets. No specific biomarkers were available to objectively verify compliance, unlike ketogenic diets where ketone bodies can be used. In addition, although the intervention aimed to create a clear dietary contrast between groups, the actual carbohydrate intake in the reduced‑carbohydrate group remained higher than the intended target and fiber intake did not significantly differ between groups at the end of the study, suggesting moderate adherence and relatively limited separation between intervention arms.

Second, analyses were conducted on a per‑protocol basis including only participants who completed the study (RCD: *n* = 29; STD: *n* = 32), and no intention‑to‑treat analysis was performed. Given the attrition rate of approximately 27%, this approach may introduce potential attrition bias and may affect the internal validity of the findings. However, baseline characteristics including age, sex, anthropometric indices, and physical activity did not differ significantly between participants who withdrew and those who completed the study, which may reduce the likelihood of substantial attrition bias.

Third, pubertal stage was not formally assessed. Considering the age range of participants (7–16 years), developmental and hormonal variability related to puberty may have influenced outcomes such as insulin resistance, lipid metabolism, body composition, and potentially gut microbiota composition. Although baseline age distribution was comparable between groups, residual confounding related to pubertal status cannot be excluded.

Finally, multiple cardiometabolic and microbiota outcomes were analyzed without formal adjustment for multiple comparisons. Therefore, the possibility of type I error cannot be ruled out and some statistically significant findings—particularly those related to exploratory microbiota analyses—should be interpreted with caution and confirmed in future studies.

In conclusion, this study suggests that a reduced‑carbohydrate diet does not provide additional benefits over a standard diet in improving anthropometric indices after 12 weeks of intervention in children and adolescents with obesity. However, the reduced‑carbohydrate diet may have favorable effects on triglyceride levels, the TG/HDL‑C ratio, and certain gut microbiota markers. Improvements in indicators such as the TG/HDL‑C ratio may reflect beneficial effects of dietary modification on aspects of cardiometabolic health. Given the exploratory nature of the microbiota analyses and the relatively small sample size, further studies with larger populations and more comprehensive microbiota assessment methods are needed to confirm these findings.

## Supplementary Information

Supplementary Material 1.

Supplementary Material 2.

Supplementary Material 3.

Supplementary Material 4.

Supplementary Material 5.

## Data Availability

The datasets analyzed during the current study are not publicly available due to institutional limitations but are available from the corresponding author upon reasonable request.

## Abbreviations

RCD: Restricted carbohydrate diet
STD: Standard diet
BMI: Body mass index
PBF: Percent body fat
WC: Waist circumference
SMM: Skeletal muscle mass
HOMA-IR: Homeostatic model assessment of insulin resistance
HDL-C: High-density lipoprotein cholesterol
LDL-C: Low-density lipoprotein cholesterol
TG: Triglycerides
GGT: Gamma-glutamyl transferase
AST: Aspartate aminotransferase
ALT: Alanine aminotransferase
