# Supplementary material for: The effect of reduced carbohydrate diet on cardiometabolic risk factors and gut microbiota in children and adolescents with obesity: a randomized controlled trial
Source: BMC Pediatr. 2026 Aug 15;26:1155. doi: 10.1186/s12887-026-07516-9 (PMC13753492; doi:10.1186/s12887-026-07516-9)
Supplement: Supplementary file 1 [file 12887_2026_7516_MOESM1_ESM.docx]

| **Supplementary Table 1**- Samples of menu on two groups of intervention | | |
| --- | --- | --- |
|  | **Reduced carbohydrate diet** | **Standard diet** |
| breakfast | 60 gr feta cheese  30 gr whole wheat bread  2 halves walnut  1 medium cucumber with peel | 60 gr white bread  30 gram feta cheese  1 date (10 gram) |
| Snack 1 | 20 almonds  1 cup low fat milk | 1 apple  1 cup low fat milk  5 cracker regular size (15 gr) |
| lunch | 70 gr cooked rice  60 gr chicken  50 gr lettuce  1 tomato  2 tbsp olive oil  0.5 cup low fat yogurt | 140 gr cooked rice  30 gram chicken  30 gram cucumber  30 gram tomato  2 tbsp olive oil  0.5 cup low fat yogurt |
| Snack 2 | 1 carrot  1 kiwi fruit  2 cooked egg whites | 1 banana  1 cup low fat milk  150 gr watermelon  1 date (10 gram) |
| Dinner | 0.5 cup cooked lentil  1tbsp chia seeds  40 gram cooked mushroom  2 cooked egg  1 tbsp olive oil  0.5 cup low fat yogurt | 1 cup pasta (180 gr)  30 gr chicken  20 gr cooked mushroom  1 small carrot  1 tbsp olive oil  0.5 cup low fat yogurt |
| Diet composition | Calorie: 1400 kcal, 30% CHO, 25% Pr, 45% fat  19 gr dietary fiber | Calorie: 1460 kcal, 58% CHO, 17% Pr, 25% fat  10 gr dietary fiber |
