# Supplementary material for: The effect of reduced carbohydrate diet on cardiometabolic risk factors and gut microbiota in children and adolescents with obesity: a randomized controlled trial
Source: BMC Pediatr. 2026 Aug 15;26:1155. doi: 10.1186/s12887-026-07516-9 (PMC13753492; doi:10.1186/s12887-026-07516-9)
Supplement: Supplementary file 2 [file 12887_2026_7516_MOESM2_ESM.docx]

**Supplementary Table 2 -** 16S rRNA gene specific primers used in study

| Target bacteria | Forward (5' to 3')^a^ | Reverse (5' to 3') | Amplicon size (bp) | References |
| --- | --- | --- | --- | --- |
| Actinobacteria | TGTAGCGGTGGAATGCGC | AATTAAGCCACATGCTCCGCT | 277 | (47) |
| Firmicutes | GGAGYATGTGGTTTAATTCGAAGCA | AGCTGACGACAACCATGCAC | 126 | (47) |
| Bacteroidetes | GTTTAATTCGATGATACGCGAG | TTAASCCGACACCTCACGG | 122 | (47) |
| Proteobacteria | AACGCGAAAAACCTTACCTACC | TGCCCTTTCGTAGCAACTAGTG | 174 | (47) |
| Verrucomicrobia | TCANGTCAGTATGGCCCTTAT | CAGTTTTNAGGATTTCCTCCGCC | 97 | (47) |
| *Prevotella* | CACCAAGGCGACGATCA | GGATAACGCCYGGACCT | 283 | (48) |
| *Lactobacillus* | AGCAGTAGGGAATCTTCCA | CACCGCTACACATGGAG | 341 | (48) |
| *Bifidobacterium* | TCGCGTCYGGTGTGAAAG | CCACATCCAGCRTCCAC | 243 | (48) |
| *Bacteroides* | GGTGTCGGCTTAAGTGCCAT | CGGAYGTAAGGGCCGTGC | 140 | (48) |
| *Escherichia* | CATTGACGTTACCCGCAGAAGAAGC | CTCTACGAGACTCAAGCTTGC | 190 | (48) |

^a^Nucleotide symbols: N = any nucleotide
