# Supplementary material for: The effect of reduced carbohydrate diet on cardiometabolic risk factors and gut microbiota in children and adolescents with obesity: a randomized controlled trial
Source: BMC Pediatr. 2026 Aug 15;26:1155. doi: 10.1186/s12887-026-07516-9 (PMC13753492; doi:10.1186/s12887-026-07516-9)
Supplement: Supplementary file 3 [file 12887_2026_7516_MOESM3_ESM.docx]

**Supplementary Table 3-** baseline characteristic of participants who dropout

|  | Remain (n=61) | Dropout (n=23) | p-value |
| --- | --- | --- | --- |
| Age, years | 11.48 ± 2.34 | 11.38 ± 2.06 | 0.847 |
| Female sex | 20 (32.8) | 10 (43.5) | 0.362 |
| Height, cm | 150.5 ± 0.12 | 150.7 ± 0.11 | 0.928 |
| height, z score | 0.57 ± 1.13 | 0.80 ± 1.07 | 0.407 |
| weight, kg | 61.2 ± 16.4 | 62.7 ± 13.0 | 0.690 |
| Body mass index, kg/m^2^ | 26.8 ± 2.7 | 26.7 ± 4.0 | 0.349 |
| body mass index, z score | 2.57 ± 0.55 | 2.77 ± 0.69 | 0.168 |
| Waist circumference, cm | 89.1 ± 11.0 | 89.0 ± 12.2 | 0.950 |
| Percent of body fat | 28.5 ± 5.5 | 30.3 ± 4.9 | 0.168 |
| Skeletal muscle mass, kg | 23.8 ± 4.7 | 23.6 ± 4.6 | 0.861 |
| Physical activity (MET/h/week) | 0.00 (0.00, 3.22) | 0.00 (0.00, 9.13) | 0.384 |
| More than 6 hour of screen time | 43 (70.5) | 20 (90.9) | 0.05 |

Continuous variables with normal distribution presented as mean ± standard deviation, continuous variables without normal distribution presented as median (interquartile Q1, Q3), categorical variables presented as frequency (percentage).

Abbreviation: BMI: body mass index
