# Supplementary material for: The effect of reduced carbohydrate diet on cardiometabolic risk factors and gut microbiota in children and adolescents with obesity: a randomized controlled trial
Source: BMC Pediatr. 2026 Aug 15;26:1155. doi: 10.1186/s12887-026-07516-9 (PMC13753492; doi:10.1186/s12887-026-07516-9)
Supplement: Supplementary file 4 [file 12887_2026_7516_MOESM4_ESM.docx]

**Supplementary Table 4-** Differences of the changes in the abundance of bacterial genera and phyla.

|  | RCD (n=22) | STD (n=23) | p-value | Effect size |
| --- | --- | --- | --- | --- |
| Actinobacteria | -0.03 ± 4.34 | -0.46 ± 4.11 | 0.73 | 0.1 |
| Firmicutes | 0.03 ± 2.42 | 0.87 ± 2.87 | 0.29 | -0.34 |
| Bacteroidetes | 0.06 ± 3.67 | 1.23 ± 4.55 | 0.35 | -0.27 |
| Proteobacteria | -0.23 ± 2.72 | 0.79 ± 2.85 | 0.22 | -0.36 |
| Verrucomicrobia | 0.52 ± 1.34 | -0.39 ± 1.33 | **0.03** | **0.68** |
| *Prevotella* | 0.30 ± 1.84 | -0.28 ± 3.26 | 0.46 | 0.23 |
| *Lactobacillus* | 0.54 ± 3.25 | -0.90 ± 4.28 | 0.21 | 0.37 |
| *Bifidobacterium* | -0.36 ± 2.80 | -0.10 ± 4.86 | 0.83 | -0.07 |
| *Bacteroides* | -0.35 ± 3.81 | 0.71 ± 4.17 | 0.37 | -0.26 |
| *Escherichia* | -0.62 ± 3.60 | -1.10 ± 3.67 | 0.66 | 0.14 |

Variables presented as mean ± standard deviation

Abbreviations: RCD: restricted carbohydrate diet, STD: standard diet
